# Identification and functional analysis of two alternatively spliced transcripts of *ABSCISIC ACID INSENSITIVE3 (ABI3)* in linseed flax (*Linum usitatissimum* L.)

**DOI:** 10.1371/journal.pone.0191910

**Published:** 2018-01-30

**Authors:** Yanyan Wang, Tianbao Zhang, Xiaxia Song, Jianping Zhang, Zhanhai Dang, Xinwu Pei, Yan Long

**Affiliations:** 1 MOA Key Laboratory on Safety Assessment (Molecular) of Agri-GMO, Institute of Biotechnology, Chinese Academy of Agricultural Sciences, Beijing, China; 2 Crop Institute, Gansu Academy of Agricultural Sciences, Lanzhou, China; National Taiwan University, TAIWAN

## Abstract

Alternative splicing is a popular phenomenon in different types of plants. It can produce alternative spliced transcripts that encode proteins with altered functions. Previous studies have shown that one transcription factor, *ABSCISIC ACID INSENSITIVE3* (*ABI3*), which encodes an important component in abscisic acid (ABA) signaling, is subjected to alternative splicing in both mono- and dicotyledons. In the current study, we identified two homologs of *ABI3* in the genome of linseed flax. We screened two alternatively spliced flax *LuABI3* transcripts, *LuABI3-2* and *LuABI3-3*, and one normal flax *LuABI3* transcript, *LuABI3-1*. Sequence analysis revealed that one of the alternatively spliced transcripts, *LuABI3-3*, retained a 6 bp intron. RNA accumulation analysis showed that all three transcripts were expressed during seed development, while subcellular localization and transgene experiments showed that *LuABI3-3* had no biological function. The two normal transcripts, *LuABI3-1* and *LuABI3-2*, are the important functional isoforms in flax and play significant roles in the ABA regulatory pathway during seed development, germination, and maturation.

## Introduction

Abscisic acid (ABA) is an important hormone that regulates many aspects of plant growth and development such as the synthesis of seed storage proteins and fatty acids[1], the promotion of drought tolerance and dormancy in seeds, the suppression of seed germination, and the transition from vegetative growth to reproductive growth [2,3]. Previous studies have shown that exogenous ABA could suppress germination of immature embryos [4–6]. Many maize mutants for ABA synthesis, including *vp2*, *vp5*, *vp7*, *vp8*, and *vp9*, have demonstrated that ABA could suppress seed germination [6]. In *Arabidopsis thaliana* and tobacco, ABA-synthesis mutants lost their dormancy characteristics, indicating that endogenous ABA could suppress seed germination and promote seed dormancy [7,8].

In the ABA signaling pathway, four key regulatory genes, including *LEAFY COTYLEDON1 (LEC1)*, *LEC2*, *FUSCA3* (*FUS3*), and *ABSCISIC ACID INSENSITIVE3* (*ABI3*) [9,10] are partially functionally redundant in the regulation of seed maturation. Of these, ABI3 is highly conserved among different plant species, including *Arabidopsis*, maize, rice, wheat, tomato, and oat [6]. In *Arabidopsis* lines over-expressing *ABI3*, expression of the seed-specific *At2S3*, *AtEM1*, *AtCRC*, *AtEM6* and *AtSOM* genes was induced by exogenous ABA, with expression in the roots and seeds found to be more sensitive to ABA treatment[11,12].

ABI3 is a transcription factor belonging to B3 domain-containing gene family. Previous studies have shown that ABI3 has four domains, A1, B1, B2, and B3, that are conserved in different plants. The A1 domain is an acidic transcriptional activator; the B1 domain is a region needed for interaction with specific bZIP transcription factors such as ABI5, bZIP10, bZIP25, and TRAB1[13]; the B2 domain can bind to either ABA response elements or the G-box element (CACGTG) and so could be involved in both transactivation or nuclear localization [14]; and the B3 domain has been shown to bind to the RY motif (CATGCA) in vitro[15].

Alternative splicing is a process that generates multiple proteins from single genes. For eukaryotes, this process is not only an important post-transcriptional regulatory system, it is also an essential mechanism for increasing transcriptome plasticity and proteome diversity. In *Arabidopsis*, approximately 42% of transcripts are alternatively spliced, with the resulting spliced transcripts encoding functionally different or non-functional proteins[16]. For example, one positive regulator of the ABA signaling pathway, *SDIR1*, has three alternative mRNA isoforms, *SDIR1-822*, *SDIR1-691*, and *SDIR1-666*, with the three isoforms having different RNA accumulation levels[17] [19]. Previous studies have found that *ABI3* is alternatively spliced in different crops such as *Arabidopsis*[18] [20], tomato [19] [21], wheat[20]], rice [21]3], and pea [22]4]. In tomato, two transcripts, *SlABI3-F* and *SlABI3-T*, were found in the genome. *SlABI3-F* encoded a full-length amino acid, while *SlABI3-T* encoded a truncated protein that lacked 30 amino acids. These two transcripts accumulated in the developing seeds and were differentially expressed at different seed development stages. This suggested that the alternative splicing resulting in these two transcripts was developmentally regulated. In wheat, McKibbin et al. (2002) found that early seed germination before harvesting was caused by the incorrect splicing of one alternatively spliced transcript, *vp1 [20]*[22]. Furthermore, many truncated *OsVP1* transcripts were found in the rice genome in plants with the same phenotype as the maize *vp1* mutant [21][23]. In the dicotyledon *Pisum sativum*, many alternatively spliced *ABI3* transcripts, *PsABI3-1–PsABI3-7*, were discovered in the genome, with sequence analysis showing that full-length *PsABI3-1* included the basic domains B1 and B3 and was expressed only in seeds [22][24].

Flax (*Linum usitatissimum* L.) is an economically significant self-pollinated crop in which the stem fiber and seed oil can both be used commercially. The seed oil and protein content are important for linseed flax; seed germination-related traits are, therefore, important in this species. In this study, *ABI3* was identified in flax, with a total of three transcripts, *LuABI3-1–3* found in the genome. Sequence analysis revealed that one of the transcripts, *LuABI3-3*, was alternatively spliced and retained a 6 bp intron. RNA accumulation analysis showed that all three transcripts were expressed during seed development, while subcellular localization and transgenic plant experiments showed that *LuABI3-3* had no biological function. The two normal transcripts, *LuABI3-1* and *LuABI3-2*, were the predominant isoforms in flax and played significant roles in the ABA regulatory pathway during seed development, germination, and maturation.

## Materials and methods

### Plant materials

Plants of the linseed flax cultivar Zhangya No.2 were grown in a greenhouse(24°C, 16h light/8h dark). The leaves of seedlings were collected for DNA extraction. When the plants flowered, siliques were collected 10, 20, 30, and 40 d after pollination (DAP); roots, stems, and leaves were also harvested for RNA extraction. The *Arabidopsis* ecotype Col-0 was used for gene transformation experiments. Nicotiana tabacum was planted for subcellular localization experiments.

### RNA isolation and cDNA synthesis

The coding sequences (CDS) of the *ABI3* transcripts were isolated from linseed flax cv. Zhangya No.2. Total RNA was extracted using the RNeasy Plant Mini Kit (Qiagen) and DNase treated (New England Biolabs) before approximately 2 μg of RNA was reverse transcribed with the oligo-dT primers to obtain first strand cDNA using a cDNA synthesis kit (Applied Biosystems).

The sequence of *Arabidopsis* ABI3 (*At3g24650*) from the NCBI database was used as the query to blast the flax genome sequence (https://phytozome.jgi.doe.gov/pz/portal.html). Two homologs of ABI3, *Lus10022820* and *Lus10011888*, were identified in the flax genome. Primers ABI3F and ABI3R (Table 1), based on the two homologs, were used to isolate the full CDS of flax *ABI3*. The polymerase chain reaction (PCR) products were cloned into the pEasy-T1 cloning vector (Transgene, China), sequenced, and analyzed using Vector NTI Advance 11 software. For each PCR product, five clones were sequenced by the Tsing Company, China.

**Table 1:** Primer sequences for *LuABI3* gene cloning and expression analysis.

Code	Primer name	Primer sequence (5'–3')
Cloning		
	ABI3 F	ATGCATGAAGAAGAAGATCTCT
	ABI3 R	TTAGACTCGGGATTTTATCTGT
	ABI3.gF	ATGCATGAAGAAGAAGATCTCTAT
	ABI3.g R	TTATCTGTATGTATCGAGTTGTTG
RNA accumulation		
1	LuABI3-1.1F	TAATCATCACAACACCGGCGT
	LuABI3-1.1R	TCCCTGCTTCTGATGGTTCTGA
2	LuABI3-2.1F	CAATCATCACACTACCGGCGC
	LuABI3-2.1R	GTCGATCCACCGTCTGCA
3	LuABI3-3.1F	TCTCAGATTCTGGCCCAACA
	LuABI3-3.1R	GCTGCCTTCTTGTTCTCAGGC
4	LuActinF	GGCATCCACGAGACCACTTA
	LuActinR	GGACCCTCCAATCCAGACAC

### DNA isolation and genomic sequence identification

As with the cDNA sequences described above, full-length genomic sequences were obtained from linseed flax cv. Zhangya No.2. Total genomic DNA was extracted from seedling leaves using the extraction method of Murray and Thompson (1980) [23]. To obtain genomic sequences, PCR amplifications were performed using the total genomic DNA and the ABI3.g F/ABI3.g R primers (Table 1). The primers were designed based on the transcript sequences. After PCR amplification, the amplicons were cloned into the pEasy-T1 cloning vector, sequenced, and analyzed using Vector NTI Advance 11 software.

### *LuABI3* expression analysis in flax tissues

Quantitative real-time PCR (qRT-PCR) analysis was used to analyze the RNA accumulation patterns of the different *LuABI3* transcripts. cDNA derived from siliques harvested 10, 20, 30, and 40 DAP, and also from roots, stems, and leaves was used. Transcript-specific primers were designed based on the transcript sequences (Table 1). The six bases “TCTCAG” were added to the 5′ end of the LuABI3-3.1F primer to specifically amplify the *LuABI3-3* fragment. Before being used in qRT-PCR, the qRT-PCR primers were first checked using normal PCR amplification and sequencing of the PCR products to confirm that the primers were transcript-specific. qRT-PCR was conducted using the ABI7500 Fast Real-time PCR system (Applied Biosystems). In our previous study, we used high-throughput sequencing technology to do RNA-seq for four tissues in four developing stages and found Actin gene could stably express in all the samples. So the *LuActin* (EU830342) gene used as a reference gene to normalize the gene expression.The efficiencies of all target genes (*LuABI3-1* to *LuABI3-3*) and Actin were determined by using a validation method as Banik described [24]. The cDNA was serially diluted (50, 25, 12.5, 6.25 and 3.125 ng) and each cDNA was amplified by real-time PCR with the gene-specific primers using the SYBR green method. Each dilution was replicated three times. The mean of three replications was used in determining the absolute value of the slope of log(input amount) versus ΔCT. For each gene, three independent PCR reactions were applied for each sample, and 2-^△△Ct^ method was used to calculate the gene relative expression values.

### Subcellular localization of LuABI3

For subcellular analysis, the complete open reading frame of the three *LuABI3* transcripts was amplified using primers 35s-ABI3-GFP -InF (5′-GACCGGTCCCGGGGGATCCATGGGAATCGACCCGTTT-3′) and 35s-ABI3-GFP-InR (5′-CCTTGCTCACCATGGATCCTCTGTATGTATCGAGTTGTTGGA-3′) that incorporated *Bam*HI restriction sites at both ends of the product. The amplified PCR fragments were cloned into the binary vector pCAMBIA1305-35s-GFP to generate 35S::LuABI3-GFP fusion constructs. These constructs were transformed into *Agrobacterium tumefaciens* strain EHA105 using the freeze–thaw method, and these transformed *Agrobacterium* strains were infiltrated into the leaves of 4- to 6-w-old tobacco plants as described by Sparkes et al. (2006). Microscopic analysis was performed 2–3 d post-infiltration using the confocal laser scanning microscope ZEISS LSM 800 system.

### Vector construction and gene transformation

To develop *LuABI3* overexpression constructs, the CDSs of the *LuABI3-1–3* transcripts were cloned into the pBinGlyRed3 vector. The plasmids were double digested with the restriction endonuclease *Eco*RI and *Xma*I and the framework was then ligated with the specific transcript fragment so that *LuABI3-1–3* expression was under the control of the CaMV 35S promoter. The constructs were transformed to *Agrobacterium* strain EHA105 using the freeze–thaw method. *Arabidopsis* Col-0 plants were then transformed using the floral dip method [25][26], with untransformed *Arabidopsis* plants used as wild-type (WT) controls. Transgenic plants were selected on MS medium supplemented with kanamycin.

### Phenotypic screening and RNA accumulation analysis of transgenic plants

For phenotypic screening, approximately 200 WT and T2 homozygous transgenic seeds were sown on 1/2 MS medium plates containing 2% sucrose and different concentrations (0, 0.3, 0.5, 1.0, 2.0, and 3.0 μM) of ABA. Three replicates were used for each line. All plates were kept in a greenhouse under standard conditions (24°C day/18°C night; 16 h light/8 h dark). Plant phenotypes were observed after 16 d growth. In addition to the phenotype screening, whole tissue of transgenic *Arabidopsis* plants treated with 2 μM ABA was harvested for RNA isolation. Total RNA was extracted using the RNeasy Plant Mini Kit (Qiagen) as previously described. The expression of the seed germination-related genes *AtEM1*, *AtEM6*, and *AtSOM* was analyzed in the transgenic *Arabidopsis* lines using primers listed in S1 Table and qRT-PCR as previously described. The expression level was normalized to the *Arabidopsis ACTIN* (*At5g62690*) control gene, and 2^−ΔΔCt^ method was used to calculate the relative expression values. Three biological replicates and two technical replicates were used for each gene.

## Results

### Identification of *LuABI3* coding and genomic sequences in linseed flax

**Fig 1:**
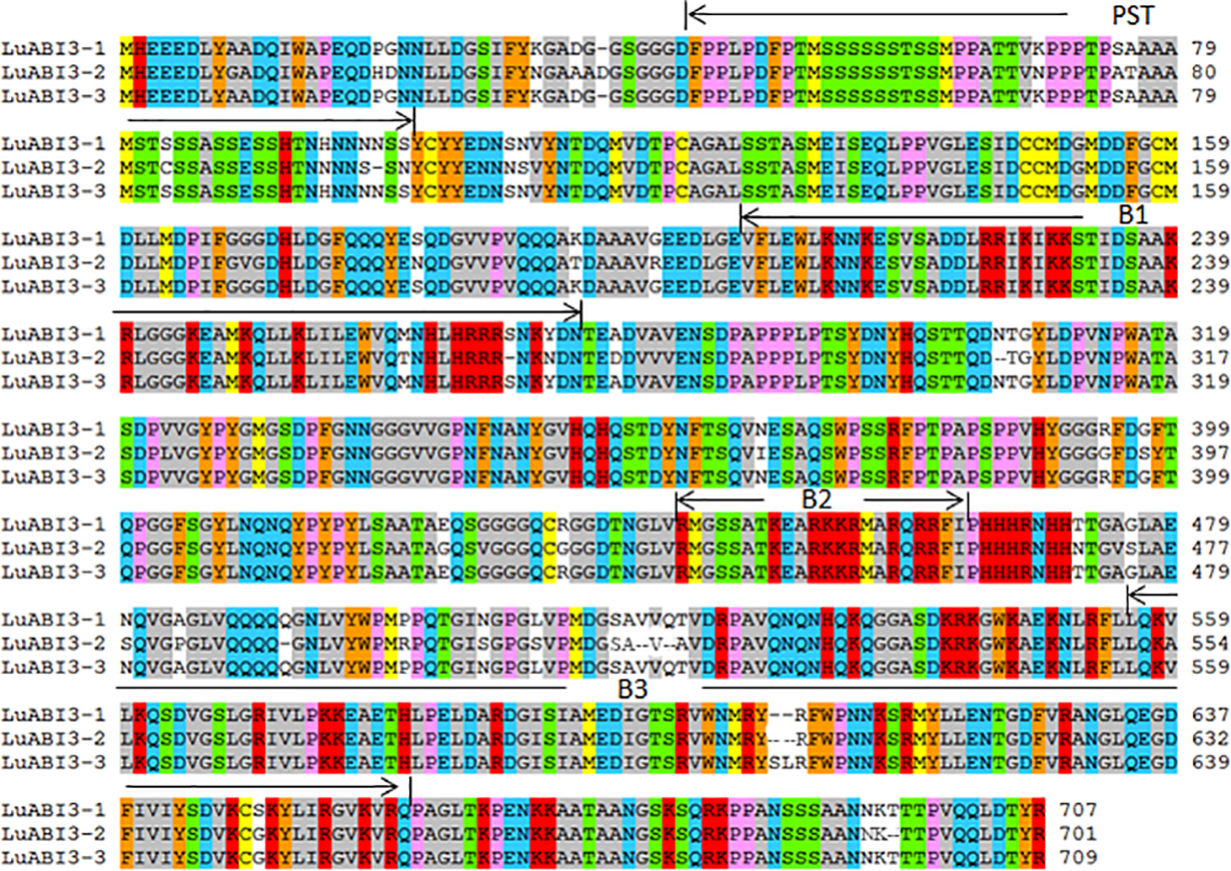
Deducing proteins comparison for LuABI3. The PST, B1, B2 and B3 represented the four domains of the LuABI3.

PCR amplification, sequencing, and sequence analysis led to the identification of three *LuABI3* CDSs. The CDSs were 2106 bp (accession number MF964255), 2124 bp (accession number MF964256), and 2030 bp (accession number MF964257) long and were named *LuABI3-1*, *LuABI3-2*, and *LuABI3-3*, respectively. Blast analysis showed that the *LuABI3-1* sequence was highly homologous to the known sequence *Lus10022820*, and *LuABI3-2* and *LuABI3-3* were highly homologous to *Lus10011888*. Primers to amplify the genomic sequences of these transcripts were designed according to the CDS. After PCR amplification and sequencing, two corresponding genomic sequences were obtained with lengths of 2661 bp and 2681 bp, respectively (accession number MF964253 and MF964254). Structural comparative analysis of the genomic and transcript sequences showed that the *LuABI3* gene had six exons and five introns; both *LuABI3-2* and *LuABI3-3* had the corresponding genomic sequence were 2681 bp long. Compared with *LuABI3-1*, *LuABI3-2* and *LuABI3-3* had three insertions in exon 1 and exon 6 (Fig 1). We also identified *LuABI3-3* transcripts with a 6 bp insertion in intron 3 when compared with *LuABI3-2*, indicating that this 6 bp intron sequence was not correctly spliced after transcription in *LuABI3-3*. We concluded, therefore, that LuABI3-2 and LuABI3-3 were alternative transcripts formed by alternative splicing.

Based on these three transcript sequences, deduced protein sequences were determined (Fig 1). The deduced proteins for LuABI3-1–3 were 701, 707, and 709 aa, respectively. Domain analysis showed that the three proteins had all four of the ABI3 conserved domains, including A1, B1, B2, and B3 (Fig 1).

### Analysis of *LuABI3* transcripts expression

**Fig 2:**
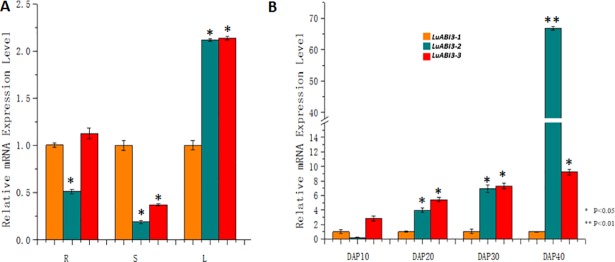
RNA accumulation analyses of three transcripts of *LuABI3*. (A) Expression pattern of different transcripts in the root (R), shoot (S), and leaf (L) of zhangya2. (B) Expression pattern of different *LuABI3* transcripts at different developmental stages.

qRT-PCR analysis was used to analyze the RNA accumulation pattern of these three transcripts. In order to ensure the similar amplification efficiencies for all the transcripts relative to the reference gene, the validation experiments were performed. The results showed that the absolute value of the slope versusΔCT was <0.1, indicating that the amplification efficiencies of Actin and all the *LuABI3* transcripts were similar. Then the expression values of all the three transcripts were calculated. It was found that the three transcripts expressed in all the tissues examined, including roots, stems, leaves, and siliques at different developmental stages (Fig 2). *LuABI3-1* was stably expressed in roots, shoots, leaves, and siliques at different developmental stages. *LuABI3-2* and *LuABI3-3* were stably expressed in roots, shoots, and leaves. In the developing siliques, *LuABI3-2* expression increased as development progressed, with the highest expression observed 40 DAP, where its expression was 66-fold higher than that of *LuABI3-1*. *LuABI3-3* expression increased from DAP10 to DAP30 and was stable from DAP30 to DAP40.

### Subcellular localization of LuABI3

**Fig 3:**
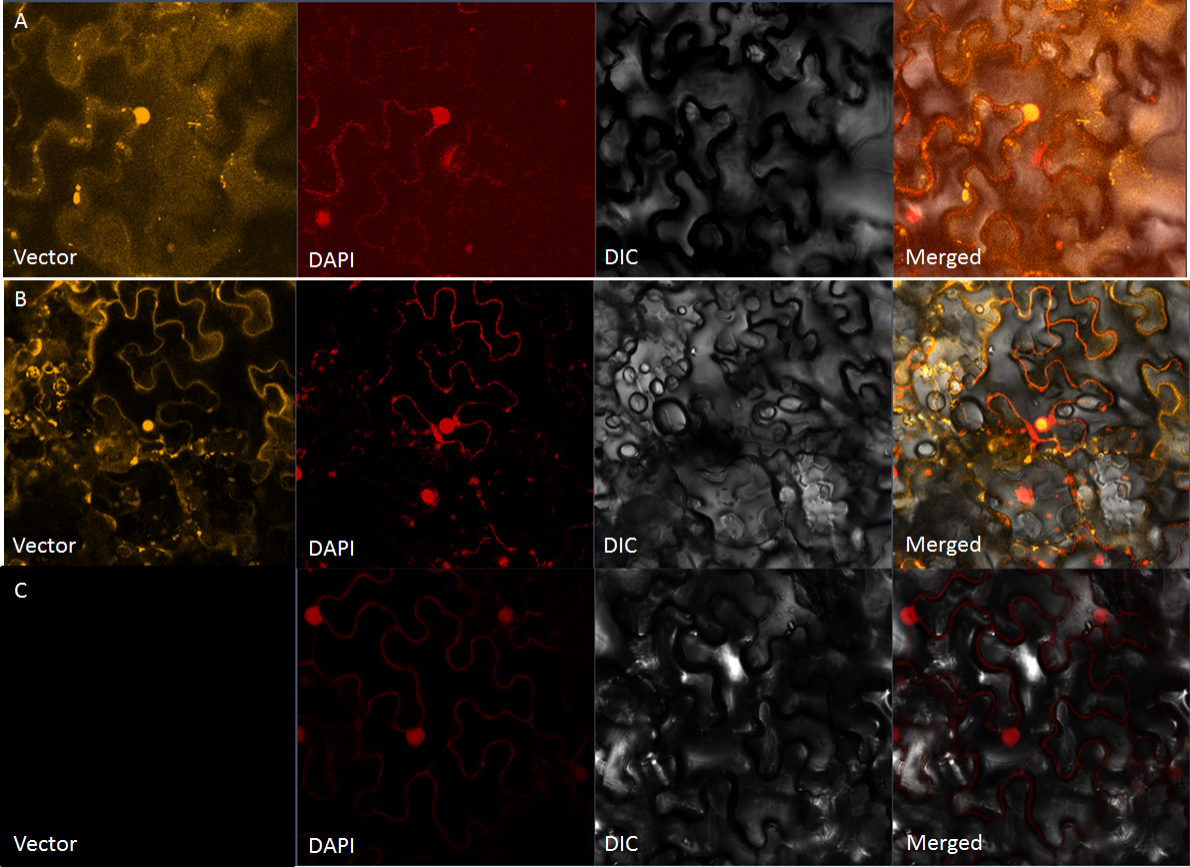
The subcellular localization results of LuABI3. (A) The subcellular localization result of LuABI3-1. (B) The subcellular localization result of LuABI3-2. (C) The subcellular localization result of LuABI3-3.

Transient expression studies in tobacco showed that constructs containing LuABI3-1 and LuABI3-2 produced a fluorescent signal. As expected, the two gene products could be detected in the cell nucleus, while the gene product of LuABI3-3 had no signal (Fig 3). This indicates that LuABI3-1 and LuABI3-2 had normal gene function in linseed flax, while LuABI3-3 was non-functional.

### Phenotypic analysis of transgenic *Arabidopsis* plants

**Fig 4:**
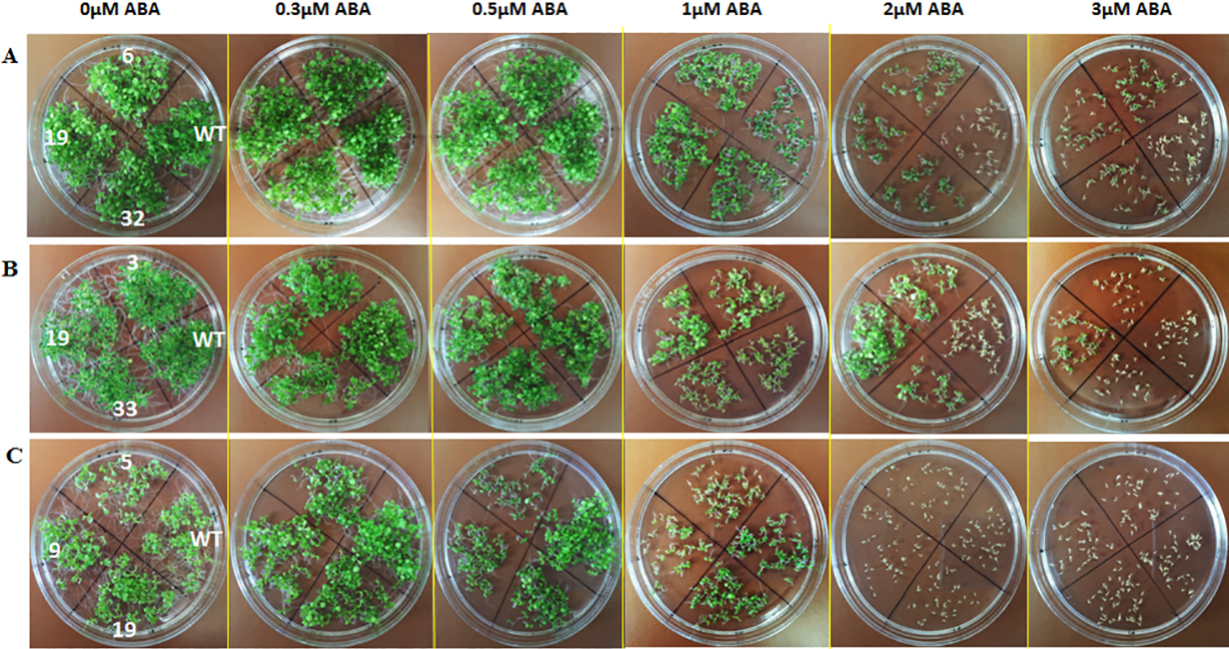
Phenotypic analysis of transgenic T2 *Arabidopsis* plants and WT treated with different concentrations of ABA. For each of the vector, seeds of three transgenic lines were selected for sowed in the petri dishes, also the seeds of WT plants were sowed. The numbers with black characters represented the three lines. The characters WT mean the wild type. (A) Phenotypic analysis of 35s:LuABI3-1 and WT plants. The three transgenic lines were line6, line19 and line32. (B) Phenotypic analysis of 35s:LuABI3-2 and WT plants. The three transgenic lines were line3, line19 and line33. (C) Phenotypic analysis of 35s:LuABI3-3 and WT plants. The three transgenic lines were line5, line9 and line19.

Both WT and transgenic plants grew normally in the ABA-free medium, and the transgenic plants grew normally in both 0.3 μM and 0.5 μM ABA medium (Fig 4). When the ABA concentration increased to 1 μM, the transgenic plants overexpressing *LuABI3-1* and *LuABI3-2* grew better than the WT plants, while for the *LuABI3-3* transgenic plants, the leaves were wrinkled and the plants were weaker than the control plants. With an ABA concentration of 2 μM, the leaves of the overexpressing *LuABI3-1* and *LuABI3-2* resulting plants were green and plant growth was inhibited.

Conversely, the germinated WT plants become yellow and then gradually died, as did the transgenic plants overexpressing *LuABI3-3*. With an ABA concentration of 3 μM, parts of the transgenic plants overexpressing *LuABI3-1* and *LuABI3-2* survived, while all the plants overexpressing *LuABI3-3* and the WT plants died. These results showed that the optimal concentration for the survival of LuABI3 transgenic plants was 2 μM and that *LuABI3-3* did not function in the ABA signaling pathway.

### Expression of seed germination-related genes in transgenic plants

**Fig 5:**
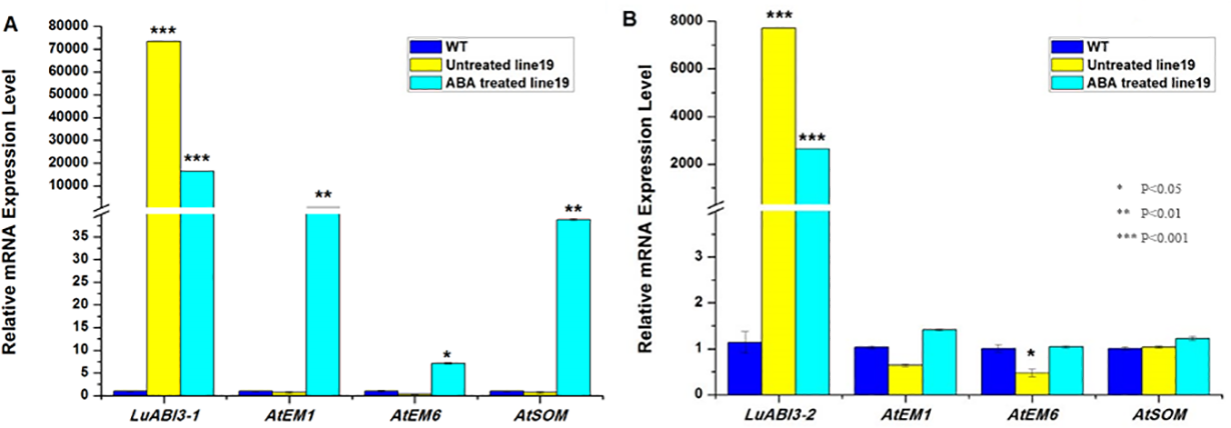
Expression analysis of three seed development-related genes in transgenic *Arabidopsis* plants. (A) Gene expression analysis in Line19 (with the 35s:LuABI3-1 construct) and WT plants. For *LuABI3-1* gene expression, the expression value was higher in the transgenic plants than that in WT plants. Expression values of *EM1*, *EM6* and *SOM* in the ABA treated plants was higher than that in untreated plants. (B) Gene expression analysis in Line19 (with the 35s:LuABI3-2 construct) and WT plants. For *LuABI3-2* gene expression, the expression value was higher in the transgenic plants than that in WT plants.

As all the transgenic plants overexpressing *LuABI3-3* died after ABA treatment, RNA accumulation analysis was done using samples from transgenic plants overexpressing *LuABI3-1* and *LuABI3-2* and the WT control plants (Fig 5). For the ABA-untreated plants, *LuABI3-1* and *LuABI3-2* were expressed more highly in the transgenic plants overexpressing *LuABI3-1* and *LuABI3* than in the WT plants, indicating that both transcripts were successfully integrated into the *Arabidopsis* plants. In the plants treated with 2 μM ABA, expression of both *LuABI3-1* and *LuABI3-*2 was lower than in the corresponding untreated plants. This indicated that exogenous ABA negatively regulated expression of *LuABI3*. Then expression of three other seed germination-related genes, *AtEM1*, *AtEM6*, and *AtSOM* were compared between the ABA treated and untreated planted for both *LuABI3-1* and *LuABI3-2* vectors. The results showed that the expression in treated plants were higher than that in the corresponding untreated plants in overexpressing *LuABI3-1* plants. And in the overexpressing *LuABI3-2* plants, the expression of the three genes didn’t have significant difficience between treated and control plants. This indicated that the expression of *AtEM1*, *AtEM6*, and *AtSOM* was positively regulated by exogenous ABA, and the *LuABI3-1* and *LuABI3-2* may have sub-functions in controlling the seed germination process.

## Discussion

As a diploid crop, flax underwent a whole-genome duplication event about 5–9 million years ago, after its divergence from poplar and castor bean[26]. That means that there are many duplicate genes in the flax genome. For example, Shivaraj et al. (2017) identified 51 aquaporin genes in the flax genome, many of which were duplicate genes [27]. In the current study, blast analysis of public flax genome data revealed the presence of two homologous *LuABI3* genes, *Lus10011888* and *Lus10022820*. Gene annotation showed that the CDS length of these genes was 2130 bp and 1125 bp, respectively. Careful analysis revealed that the assembled *Lus10022820* CDS lacked the upstream sequence. Next, two actual *LuABI3* genes were identified in flax genome, with homology analysis showing that these two genes were associated with *Lus10011888* and *Lus10022820*, respectively. Structural analysis showed that, like the gene structure of *ABI3* in *Arabidopsis*, the two flax homologous genes had 6 exons and 5 introns, including the A1, B1, B2 and B3 domains and the PST domain. These four domains have been confirmed to be conserved in members of the ABI3/VP1 subfamily of the B3-domain protein family [28]. Comparison of the two genomic and three transcript sequences revealed that the three transcripts could be divided into two groups, corresponding to the two genomic sequences.

Previous studies showed that alternative splicing commonly exists in ABI3 in different crops. For example, two splicing isoforms, ABI3-ɑ and ABI3-ß, have been found *Arabidopsis* [29], while SlABI3-F and SlABI3-T have been found in tomato [19]. We hypothesized, therefore, that *LuABI3-2* and *LuABI3-3* were alternatively spliced transcripts, with *LuABI3-3* retaining a 6 bp intron. The mechanism of splicing was, therefore, intron retention. Previous studies have shown that there are four types of alternative splicing events in *Arabidopsis*: exon skipping/inclusion, an alternative 5’ splice site, an alternative 3’ splice site and intron retention. Of these, intron retention was the most frequent type, responsible for up to 40% of the alternatively spliced transcript in the genome[30]. Many alternatively spliced transcripts in different crops are non-functional, including *SlABI3-T* in tomato[19]. This lack of functionality is often a result of non-functional protein isoforms, such as truncated proteins, formed from alternatively spliced transcripts with frameshifts resulting in premature stop codons.

The RNA accumulation results obtained in this study showed that *LuABI3-1–3* were expressed in a range of different tissues, including roots, stems, leaves, and developing seeds. It is important for choosing suitable reference gene for qRT-PCR analysis to detect the RNA accumulation. In previous studies, different researchers selected different gene as reference gene in flax. For example, Huis found that *GADPH* and 2 *TEF* genes could be used as reference genes for evaluating RNA accumulation values based on different analysis methods [31].While Fernart et al., selected_c3168 and c10916 as reference genes based on their micro array analysis [32]. In the current study, we selected *Actin* gene as a reference gene because it was found that Actin could stably express in four tissues from four developing stages by using high-through put sequencing technology. After selecting reference genes, the PCR effiencies were detected first to confirm the consistent PCR amplification for target genes and reference gene. The qRT-PCR experiments showed that three transcripts had different expression patterns in developing seeds, with *LuABI3-2* having much higher expression than *LuABI3-1*. This suggests that these two transcripts may have different sub-functions during seed development. This is consistent with different homologous genes having sub-functions in regulating one specific biological process, particularly in polyploid plants. For example, there are, generally, six homologous genes in the genome of a polyploid crop plant such as *Brassica napus* compared with the model plant *Arabidopsis* because of the polyploidization process; Zou et al. (2012) identified six *BnFLC* homologs in *B*. *napus* genome. RNA accumulation experiments using these homologs showed that each had distinct expression patterns in different organs at different developmental stages [33]. Although the alternatively spliced *LuABI3-3* transcript was expressed in different tissues, the subcellular localization and transgenic plant experiments showed that this transcript had no biological function.

ABI3 is a core regulator of the ABA signaling pathway. It has been confirmed that exogenous ABA can mediate ABI3 degradation via several regulators, allowing seeds to germinate [11,34]. For example, Gao et al., (2014) identified two wheat AIP2 genes that could negatively regulate ABI3 and ABI5 in the ABA signaling pathway and were found to have important roles in seed germination [34]]. To dissect the biological function of *LuABI3*, the expression of *LuABI3-1* and *LuABI3-2* was examined in transgenic plants overexpressing *LuABI3-1* and *LuABI3-2* and WT control plants. Their expression was consistently lower in ABA-treated plants than in plants without ABA treatment. This result was consistent with previous studies and suggests that the flax ABI3 genes are sensitive to exogenous ABA and that their encoded proteins may be degraded with ABA treatment. Meanwhile, three seed germination-related genes *AtEM1*, *AtEM6*, and *AtSOM*, were more highly expressed in the transgenic plants with ABA treatment than without ABA treatment. Overall, these results demonstrate that the *ABI3* genes *LuABI3-1* and *LuABI3-2* in linseed flax function in regulating seed germination and dormancy and that the expression of these genes is dependent on ABA and independent of these two *LuABI3* genes.

## Supporting information

S1 Table Primer sequences for expression analysis of seed development related genes. (XLSX)
